# A compact and mobile hybrid C-arm scanner for simultaneous nuclear and fluoroscopic image guidance

**DOI:** 10.1007/s00330-021-08023-4

**Published:** 2021-06-16

**Authors:** Martijn M. A. Dietze, Britt Kunnen, Frank Brontsema, Pascal Ramaekers, Casper Beijst, Maryam Afifah, Arthur J. A. T. Braat, Marnix G. E. H. Lam, Hugo W. A. M. de Jong

**Affiliations:** 1 Radiology and Nuclear Medicine, University Medical Center Utrecht and Utrecht University, P.O. Box 85500, 3508 GA Utrecht, The Netherlands; 2 Image Sciences Institute, University Medical Center Utrecht and Utrecht University, P.O. Box 85500, 3508 GA Utrecht, The Netherlands

**Keywords:** Hybrid imaging, Scintigraphy, Fluoroscopy, SPECT, Cone-beam CT

## Abstract

**Purpose:**

This study evaluates the performance of a mobile and compact hybrid C-arm scanner (referred to as IXSI) that is capable of simultaneous acquisition of 2D fluoroscopic and nuclear projections and 3D image reconstruction in the intervention room.

**Results:**

The impact of slightly misaligning the IXSI modalities (in an off-focus geometry) was investigated for the reduction of the fluoroscopic and nuclear interference. The 2D and 3D nuclear image quality of IXSI was compared with a clinical SPECT/CT scanner by determining the spatial resolution and sensitivity of point sources and by performing a quantitative analysis of the reconstructed NEMA image quality phantom. The 2D and 3D fluoroscopic image of IXSI was compared with a clinical CBCT scanner by visualizing the Fluorad A+D image quality phantom and by visualizing a reconstructed liver nodule phantom. Finally, the feasibility of dynamic simultaneous nuclear and fluoroscopic imaging was demonstrated by injecting an anthropomorphic phantom with a mixture of iodinated contrast and ^99m^Tc.

**Conclusion:**

Due to the divergent innovative hybrid design of IXSI, concessions were made to the nuclear and fluoroscopic image qualities. Nevertheless, IXSI realizes unique image guidance that may be beneficial for several types of procedures.

**Key Points:**

*• IXSI can perform time-resolved planar (2D) simultaneous fluoroscopic and nuclear imaging.*

*• IXSI can perform SPECT/CBCT imaging (3D) inside the intervention room.*

**Supplementary Information:**

The online version contains supplementary material available at 10.1007/s00330-021-08023-4.

## Introduction

To date, imaging in the intervention room has primarily consisted of using fluoroscopy for mapping of the anatomy or using nuclear imaging for localization of tumors and potential metastasis. Following the success of diagnostic hybrid imaging, we believe that there may be added value in combining these two interventional modalities: i.e., performing fluoroscopy simultaneously with nuclear imaging. Such a hybrid modality would impact two types of procedures.

Firstly, the procedures that are currently done under nuclear imaging guidance but may benefit from additional fluoroscopic imaging. These procedures include sentinel node procedures [1] and parathyroidectomies [2]. Simultaneous acquisition of anatomical information in these procedures would improve the interpretability of the nuclear images. This may realize a similar benefit as is achieved in diagnostic hybrid imaging (e.g., SPECT/CT, PET/CT).

Secondly, the procedures that are currently done under fluoroscopic image guidance but may benefit from additional nuclear imaging. For instance, radioactive particles are injected through a catheter in intra-arterial peptide receptor radionuclide therapy [3] and radioembolization [4, 5]. Simultaneous acquisition of nuclear imaging in these procedures will provide direct feedback on the acquired activity distribution, which improves the safety and the efficacy. Additional nuclear imaging may furthermore aid myocardial interventions [6] by providing complementary information for the localization of a defect.

We have previously proposed an innovative detector assembly that realizes hybrid nuclear and fluoroscopic imaging in the intervention room and evaluated this design through prototype systems [7–9]. The aim of the current study is to evaluate the imaging performance of the first scanner configuration that has been approved by the local ethics committee to be evaluated in existing clinical workflows.

## Materials and methods

### IXSI

**Fig. 1:**
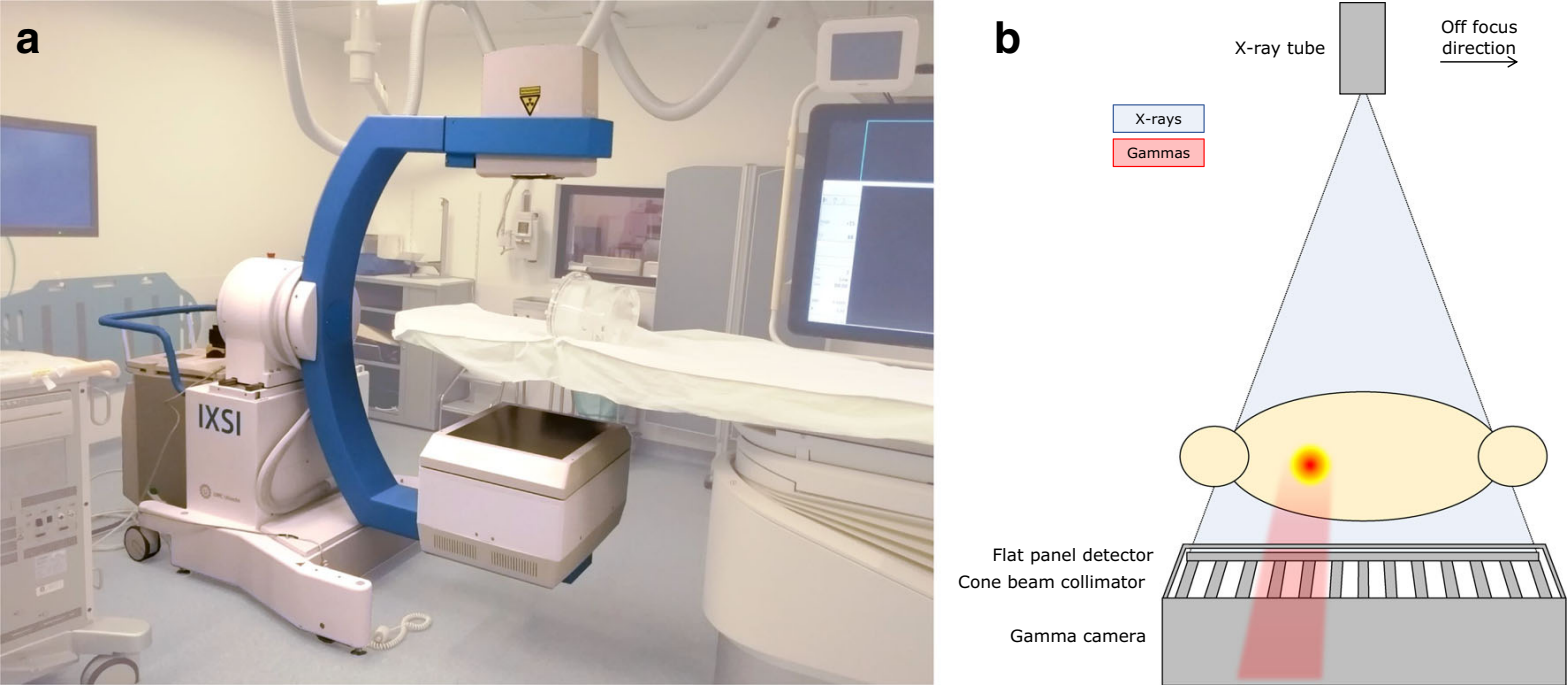
**a** IXSI in the intervention room. **b** A schematic illustration of the IXSI scanner components. For the in-focus configuration, the collimator holes are aligned with the x-ray focal point. For the off-focus configuration, the x-ray tube is shifted by 5 cm in the indicated direction

The developed scanner will be referred to as *IXSI* (Interventional X-ray and Scintigraphy Imaging). The detector stack of IXSI (see Fig. 1a) consists of an x-ray flat panel detector (Pixium 3040; Trixell) that is positioned in front of a gamma camera (P3000; Inter Medical) that is mounted with a cone-beam collimator (Nuclear Fields). The detector stack is placed on a custom-made C-arm (Indes) together with an x-ray tube (Veradius; Philips Healthcare) on the opposite side. The x-ray tube is placed in the focal point of the cone-beam collimator (105 cm), which results in simultaneously overlapping nuclear and fluoroscopic projections (see Fig. 1b). The C-arm can translate in two dimensions while rotating and can perform parameterized non-circular orbits. A collision detector is present on top of the detector stack to ensure movement termination upon contact with an object.

The flat panel detector module was adjusted from the commercial product by the reduction of the back-shielding thickness and the removal of highly attenuating objects (e.g., support screws). This adjusted flat panel detector module has a thickness of 6.8 cm, an average gamma transmission of 52% at 140 keV, and a FOV of 39.9 × 29.5 cm^2^. Different from a conventional CBCT scanner was that no anti-scatter grid was present in front of the flat panel detector to increase the gamma photon transmission rate to the gamma camera.

The cone-beam collimator has holes with 40.0-mm length, 1.90-mm inner diameter, and 0.25-mm septal thickness. The gamma camera has a 9.5-mm-thick NaI(Tl) crystal, a 3.9-mm full width at half maximum (FWHM) intrinsic spatial resolution, a 9.4% energy resolution (at 140 keV), and a 51.0 × 38.1 cm^2^ effective FOV.

The majority of the x-ray photons is absorbed by the flat panel detector when imaging simultaneously. However, a fraction penetrates the flat panel detector to be absorbed by the gamma camera, possibly deteriorating the gamma camera performance [10, 11]. In this work, the x-ray tube was translated off-focus from the focal point of the cone-beam collimator by 5 cm (see Fig. 1b). This off-focus configuration introduces a small distance-dependent mismatch between the fluoroscopic and nuclear projections but greatly reduces the nuclear and fluoroscopic interference. These effects are described in greater detail in the supplementary material.

### Nuclear image quality

The nuclear image quality of IXSI was compared to a clinical SPECT/CT scanner (Symbia T16; Siemens Healthineers). The clinical scanner had two detector heads that were mounted with parallel-hole low-energy high-resolution (LEHR) collimators (24.05-mm hole length, 1.11-mm inner diameter, and 0.16-mm septal thickness) and had a 9.5-mm-thick NaI(Tl) crystal, a 3.9-mm FWHM intrinsic spatial resolution, a 9.9% energy resolution (at 140 keV), and a 53.3 × 38.7 cm^2^ effective FOV.

#### Planar imaging

A ^99m^Tc point source (30 MBq) was positioned at distances from 5 to 40 cm to the detector (in steps of 5 cm) and measured for 1 min. The detector spatial resolution was determined by measuring the FWHM of the Gaussian functions fitted to the projections (for IXSI, divided by the projection magnification from the cone-beam collimator). The detector sensitivity was determined by dividing the total obtained counts by the calibrated source activity.

#### SPECT

The NEMA image quality phantom (150 MBq ^99m^Tc total activity; the spheres filled at an 8:1 concentration ratio) was scanned on both IXSI and the clinical scanner for 10 min over 360° with 120 angles. Both scanners rotated closely around the phantom. The scans were repeated 5 times (with the acquisition times adjusted to account for radioactive decay) to assess the stability of the reconstructions.

The nuclear projections were, for both scanners, reconstructed with the Utrecht Monte Carlo System iterative reconstruction software package, which includes attenuation compensation, resolution recovery, and Monte Carlo–based scatter correction [12, 13]. The projections were reconstructed on a 128 × 128 × 128 grid with a 4.79-mm isotropic voxel size. The reconstructor used the OSEM reconstruction algorithm (6 iterations with 8 subsets) and the reconstructions were smoothed with a 5-mm FWHM Gaussian filter. For the clinical scanner, the attenuation map was retrieved from the clinical software after performing a low-dose CT (made with 110 kVp at 14 mAs). For IXSI, the clinical attenuation map was retrieved by registering the CBCT reconstruction (made with 80 kVp at 73 mAs) to the attenuation map of the clinical scanner using the elastix software package [14].

The activity recoveries of the spheres in the NEMA image quality phantom (delineated on the low-dose CT reconstruction) were determined as a measure of the reconstruction quality.

### Fluoroscopic image quality

The fluoroscopic image quality of IXSI was compared to a clinical CBCT scanner (Allura Xper FD20; Philips Healthcare). The flat panel detector of the clinical scanner had a FOV of 37.4 × 28.7 cm^2^ and was equipped with an anti-scatter grid.

#### Planar imaging

An image quality phantom (Fluorad A+D; Pehamad) was imaged with fluoroscopy on both scanners. Both acquisitions were performed using 80 kVp and 2 mAs.

#### CBCT

An image quality phantom designed for low-contrast liver imaging (Liver Nodule Phantom; QRM) was scanned on both scanners. For IXSI, the acquisition was performed using 80 kVp and 73 mAs (the same settings that will be used in the planned clinical radioembolization study). For the clinical scanner, the acquisition was performed using 119 kVp and 504 mAs (the same settings that are clinically used for abdomen imaging). The CBCT reconstruction of the clinical scanner was retrieved from the clinical software. The CBCT reconstruction of IXSI was made using the Astra toolbox software package [15] in combination with an image-based scatter correction [16].

### Simultaneous planar imaging

Finally, the potential for simultaneous fluoroscopic and nuclear imaging was demonstrated. An empty syringe of 20 ml was placed in an anthropomorphic thorax phantom (Radiology Support Devices) and during 40 s filled with a mixture of iodinated contrast and 50 MBq ^99m^Tc via a connecting tube. The fluoroscopic projections were acquired at 80 kVp (27.8 ms pulse length, 3.75 Hz, 1.17 mA). The nuclear projections were created with an integration time of 2.7 s and were smoothed with a 5-mm FWHM Gaussian filter. Digital subtraction images were created by subtracting the fluoroscopic projections obtained during the injection with those acquired before the start of the injection.

## Results

### Nuclear image quality

#### Planar imaging

**Fig. 2:**
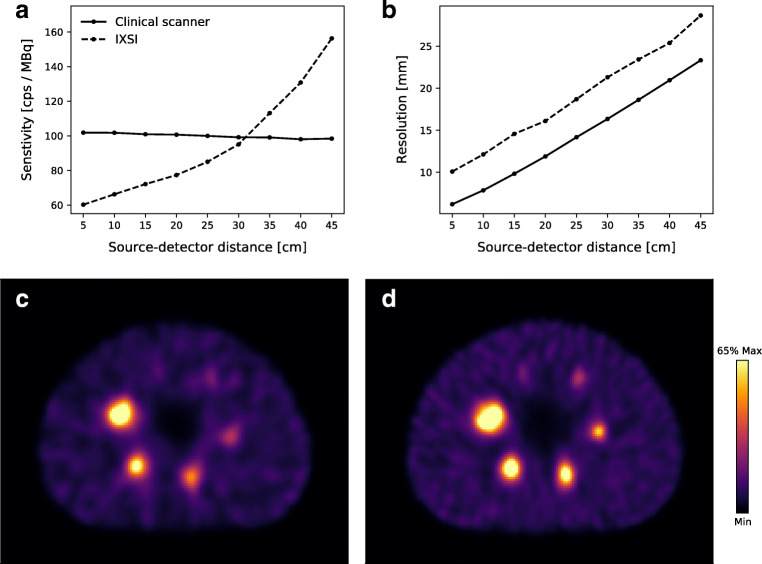
The nuclear image quality evaluation. **a** The detector sensitivity and (**b**) the spatial resolution, as a function of the distance from the source to the detector, for IXSI and one detector head of the clinical scanner. **c** The SPECT reconstruction from IXSI. **d** The SPECT reconstruction from the clinical scanner

The detector sensitivity and resolution, as a function of the distance of the point source to the detector, are collected in Fig. 2a and b for IXSI and the clinical scanner. IXSI had a lower sensitivity than the clinical scanner due to the increased attenuation from the flat panel detector module, but increased over the source to detector distance due to the converging nature of the cone-beam collimator. IXSI had a worse spatial resolution than the clinical scanner (consistently ~5 mm larger) due to the extra source to collimator distance arising from the thickness of the flat panel detector module.

#### SPECT

Slices of the SPECT reconstructions of the NEMA image quality phantom are shown in Fig. 2c and d. The clinical scanner acquired (5.38 ± 0.02) × 10^6^ nuclear counts, while IXSI acquired (2.25 ± 0.01) × 10^6^ counts. The activity recoveries of the NEMA image quality phantom spheres are given in Table 1. The activity recoveries from IXSI showed < 8% difference compared with those obtained by the clinical scanner.

**Table 1:** The activity recoveries (in percentage) of the NEMA image quality phantom spheres for IXSI and the clinical scanner

Sphere diameter (mm)	IXSI	Clinical scanner
37	72.7 ± 0.9	74.2 ± 0.9
28	61.7 ± 1.1	68.2 ± 1.0
22	50.4 ± 4.7	53.3 ± 2.1
17	37.2 ± 2.8	44.5 ± 4.8
13	25.6 ± 2.9	31.2 ± 1.7
10	17.9 ± 2.5	18.8 ± 2.1

### Fluoroscopic image quality

#### Planar imaging

**Fig. 3:**
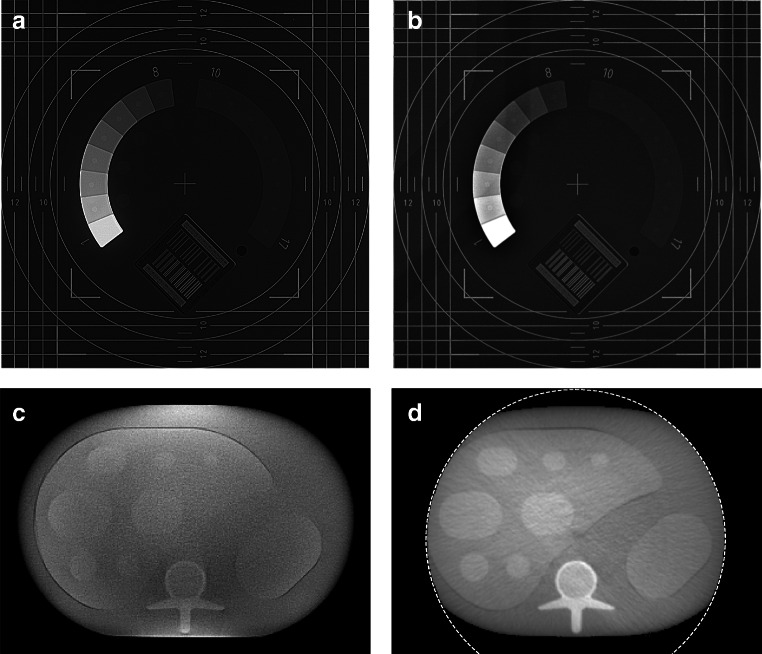
The fluoroscopic image quality evaluation. **a** The planar fluoroscopic acquisition from IXSI. **b** The planar fluoroscopic acquisition from the clinical scanner. **c** The CBCT reconstruction from IXSI. **d** The CBCT reconstruction from the clinical scanner. The white dashed lines indicate the reconstruction field of view

The planar images obtained through fluoroscopic imaging are shown in Fig. 3a and b. Visually, the images provided a comparable linearity, contrast, and resolution.

#### CBCT

Slices of the CBCT reconstructions of the liver nodule phantom are shown in Fig. 3c and d. IXSI reconstructed a larger field of view than the clinical scanner because IXSI performed a non-circular orbit instead of a circular one. The reconstruction from the clinical scanner provided a better subjective image quality in terms of contrast and uniformity than the IXSI reconstruction because it had fewer scattered photons present in the projections due to the higher kVp and mAs setting and the incorporated anti-scatter grid.

### Simultaneous planar imaging

**Fig. 4:**
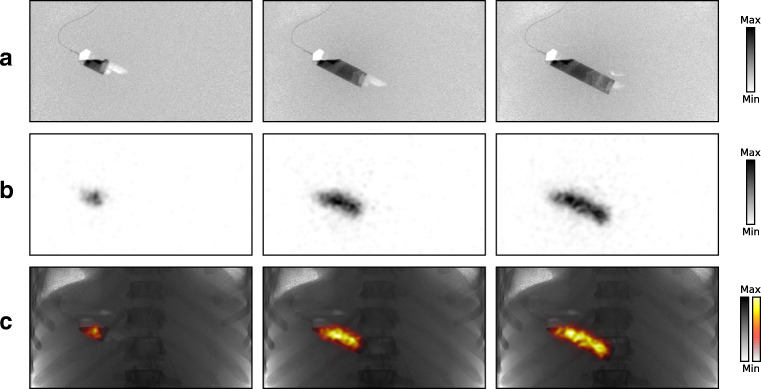
The projections at three time points (15, 30, and 40 s from the start of the injection) that are obtained during the injection of iodinated contrast with ^99m^Tc. Shown are (**a**) the digital subtraction images, (**b**) the nuclear projections, and (**c**) the fluoroscopic projections (in gray-scale) merged with the nuclear projections (in color)

The nuclear, fluoroscopic, and digital subtraction projections obtained during the injection of iodinated contrast with ^99m^Tc are shown in the supplemental material. Three timeframes are shown in Fig. 4.

## Discussion

This study evaluated the imaging capabilities of a novel hybrid C-arm scanner that has been approved by the local ethics committee to be evaluated in existing clinical workflows. The combination of x-ray and nuclear detection capability in one detector allows for a compact scanner that is compatible with a C-arm which enables a smooth implementation in the intervention room.

The phantom experiments showed that the SPECT image quality of IXSI was lower than that of a clinical scanner due to the placement of the flat panel detector in front of the gamma camera and the change from two to one detector head. However, in the case of imaging during radioembolization, it has through digital and experimental studies [9, 17–19] been demonstrated that IXSI can be expected to accurately retrieve various clinically and dosimetric important measures. The clinical performance of IXSI in other procedures is under active investigation.

The phantom experiments showed that the CBCT image quality of IXSI was lower than that of a clinical scanner due to scattered photons. For now, the obtained quality is sufficient since we focus on the addition of nuclear images to clinical workflow. In a later stage of the project, we aim to implement the following: (i) a focused anti-scatter grid to reduce the number of scattered photons is reduced, and (ii) a dedicated scatter compensation algorithm in the CBCT reconstruction.

The scanner will first be employed during the pretreatment procedure of hepatic radioembolization. Simultaneous planar imaging will be used to learn about the potential dynamic uptake behavior of ^99m^Tc-macroaggregated albumin (MAA) particles and a SPECT/CBCT scan will be made to determine dosimetric measures (e.g., the lung-shunting fraction and tumor uptake ratios). With such interventional imaging, it may become possible to merge the pretreatment procedure and the subsequent treatment in a single-session procedure. This innovation could lead to more personalized treatments and will allow for one-stop-shop workflows.

Concluding, a novel hybrid scanner shows promising imaging capabilities for aiding interventions involving radionuclides. Clinical studies are now required for a full evaluation of the device.

## Supplementary information

ESM 1 The projections that are obtained live during the injection of iodinated contrast with ^99m^Tc. Shown are (left) the digital subtraction images, (middle) the nuclear projections, and (right) the fluoroscopic projections (in gray-scale) merged with the nuclear projections (in color). (GIF 34.9 mb)

ESM 2 The influence of the off-focus geometry on the nuclear and fluoroscopic interference. (DOCX 257 kb)

## Abbreviations

CBCT: Cone-beam computed tomography
CT: Computed tomography
FOV: Field of view
FWHM: Full width at half maximum
IXSI: Interventional x-ray and scintigraphy imaging
SPECT: Single-photon emission computed tomography

## Declarations

### Guarantor

The scientific guarantor of this publication is Hugo WAM de Jong.

### Statistics and Biometry

No complex statistical methods were necessary for this paper.

### Methodology

• experimental

• performed at one institution
