# Supplementary figures and images for: A compact and mobile hybrid C-arm scanner for simultaneous nuclear and fluoroscopic image guidance
Source: Eur Radiol. 2021 Jun 16;32(1):517–23. doi: 10.1007/s00330-021-08023-4 (PMC8660732; doi:10.1007/s00330-021-08023-4)

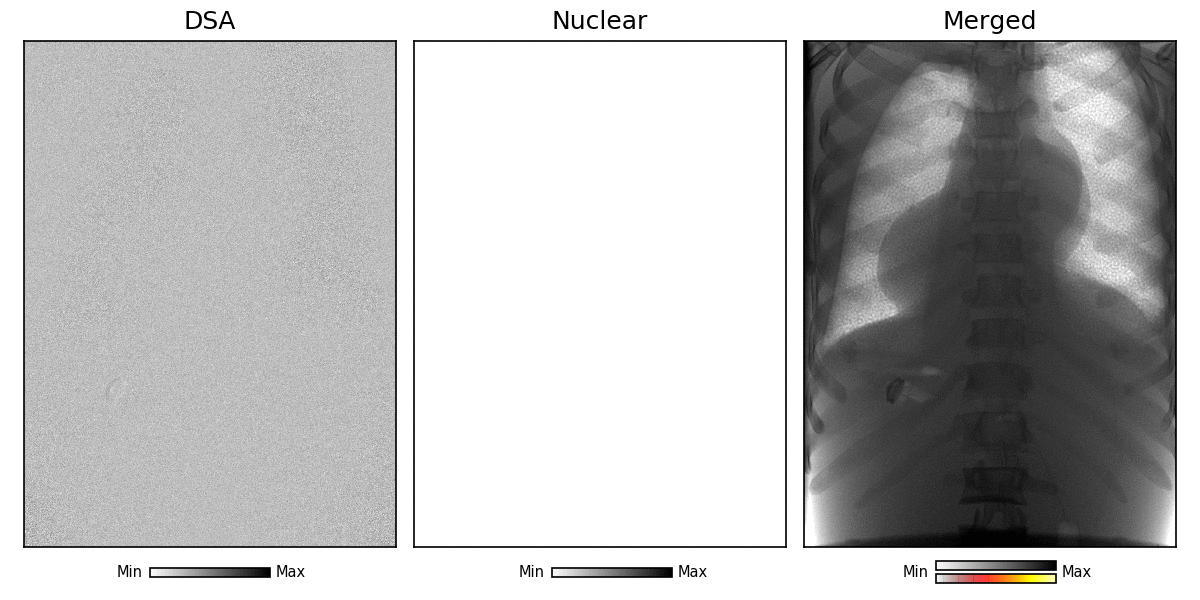

Supplement: Supplementary file 1 — The projections that are obtained live during the injection of iodinated contrast with 99mTc. Shown are (left) the digital subtraction images, (middle) the nuclear projections, and (right) the fluoroscopic projections (in gray-scale) merged with the nuclear projections (in color). (GIF 34.9 mb) [file 330_2021_8023_MOESM1_ESM.gif]
