# Supplementary material for: A compact and mobile hybrid C-arm scanner for simultaneous nuclear and fluoroscopic image guidance
Source: Eur Radiol. 2021 Jun 16;32(1):517–23. doi: 10.1007/s00330-021-08023-4 (PMC8660732; doi:10.1007/s00330-021-08023-4)
Supplement: Supplementary file 2 — The influence of the off-focus geometry on the nuclear and fluoroscopic interference. (DOCX 257 kb) [file 330_2021_8023_MOESM2_ESM.docx]

**Nuclear and x-ray interference**

The majority of the x-ray photons is absorbed by the flat panel detector when imaging simultaneously. However, a fraction penetrates the flat panel detector to be absorbed by the gamma camera. Previously, it was demonstrated that this x-ray spillover effect does not influence the gamma camera performance for dose levels of up to 80 kVp (at 1.17 mA) [1, 2]. However, since the fluoroscopic protocols in our institute are often performed with higher beam strengths, a higher maximum tolerable beam strength may be beneficial.

In this work, we propose to translate the x-ray tube off-focus from the focal point of the cone-beam collimator. Such a translation introduces a distance-dependent mismatch between the fluoroscopic and nuclear projections (due to geometrical effects) but has the advantage that x-ray spillover is greatly reduced (due to additional attenuation from the SPECT collimator).

The impact positioning the x-ray tube off-focus from the focal point of the cone-beam collimator on the x-ray transmission was studied using simulations with the GATE software package [3]. In the simulation, a point source of 80 keV (a relatively high approximation of the mean x-ray energy) was positioned in the focal point of the cone-beam collimator of IXSI and hence translated off-focus in steps of 5 mm. The relative transmission of the x-rays photons was calculated for every off-focus distance. These results are shown in Fig. 1.

The relative transmission of the x-ray photons gradually decreased between 0 and 5 cm, owing to the increased attenuation by the cone-beam collimator septa. With off-focus distances of > 5 cm, the direct transmission through the collimator holes is completely covered and only scattered x-ray photons pass through the collimator. This fraction is < 1% of the original x-ray transmission.

The optimal value for the off-focus distance is ideally as small as possible (to limit geometrical registration effects) but great enough to substantially decrease the x-ray photon flux. Given the results from Fig. 1, we believe that the optimal value for our configuration is achieved at an off-focus distance of 5 cm. Two experiments were conducted to study the practical implications of this configuration.

*X-ray spillover*A 350 MBq ^99m^Tc point source was placed on the back of an anthropomorphic thorax phantom (Radiology Support Devices) to create a realistic scatter situation. Nuclear projections were acquired in list-mode and the gamma count rate in the 140 ± 7.5% keV energy window was determined. Fluoroscopic projections (27.8 ms pulse length, 3.75 Hz) were then acquired from 70 to 100 kVp (1.14 to 1.22 mA) while the gamma count rate in the same energy window was again determined (for the intervals in which the x-ray tube was not active). The usable gamma data fraction, as a function of the x-ray tube strength, was calculated by dividing these two count rates. This measurement was done for both the in-focus and the off-focus configuration.

The usable data fraction of the gamma camera is for both configurations shown in Fig. 2b as a function of the x-ray tube beam strength. For the in-focus configuration, substantial gamma data loss (< 95% useable data) occurred for x-ray tube strengths of ≥ 85 kVp. For the off-focus configuration, no substantial gamma data loss was observed for the studied x-ray tube voltages. This experiment confirms the results from the simulation study that the x-ray flux is substantially reduced for the off-focus configuration, resulting in a higher tolerable x-ray tube strength.

*Registration mismatch*Three ^57^Co pen markers (1.2 MBq each) were positioned 5, 15, and 25 cm in front of the detector stack. Nuclear projections (measured for 2 minutes and smoothed with a 5 mm FWHM Gaussian filter) and fluoroscopic projections were acquired with both the in-focus and the off-focus configuration. The centers of the pen markers were determined in both projections and the mismatch was quantified.

The fluoroscopic and nuclear projections of the point sources obtained with the in- and off-focus x-ray tube configuration are shown in Fig. 2a. The off-focus geometry introduced a distance-dependent mismatch between the fluoroscopic and the nuclear projections due to geometrical effects. One can choose at what object distance perfect overlap of the fluoroscopic and nuclear projections occurs by translating one projection with respect to the other. If one e.g. chooses to overlap at 5 cm from the detector, offsets will be present for features at 15 and 25 cm from the detector. If one chooses to overlap the projections at 15 cm from the detector, offsets will be present for features at 5 and 25 cm from the detector. In this work, the overlap was set to a distance of 15 cm from the detector since we believe this will be close to the average distance of activity depositions to the detector in radioembolization distributions. For this reason, the projections overlap at the pen marker at 15 cm distance in Fig. 2a. The resulting offset for the pen markers positioned at 5 and 25 cm from the detector was < 1 cm.

The mismatch between the nuclear and fluoroscopic projections may limit the simultaneous image guidance of small activity accumulations. However, when imaging relatively larger accumulations (e.g. in the planar imaging phantom experiment in the main manuscript), the average mismatch will be comparable with the collimator resolution and the interpretation of the projections will not be substantially affected.

*References*

1. Koppert WJC, van der Velden S, Steenbergen JHL, de Jong HWAM (2018) Impact of intense x-ray pulses on a NaI(Tl)-based gamma camera. Phys Med Biol 63:065006. https://doi.org/10.1088/1361-6560/aaaf02

2. Koppert WJC, Dietze MMA, van der Velden S, et al (2019) A comparative study of NaI(Tl), CeBr3, and CZT for use in a real-time simultaneous nuclear and fluoroscopic dual-layer detector. Phys Med Biol 64:135012. https://doi.org/10.1088/1361-6560/ab267c

3. Jan S, Santin G, Strul D, et al (2004) GATE: a simulation toolkit for PET and SPECT. Phys Med Biol 49:4543–4561. https://doi.org/10.1088/0031-9155/49/19/007


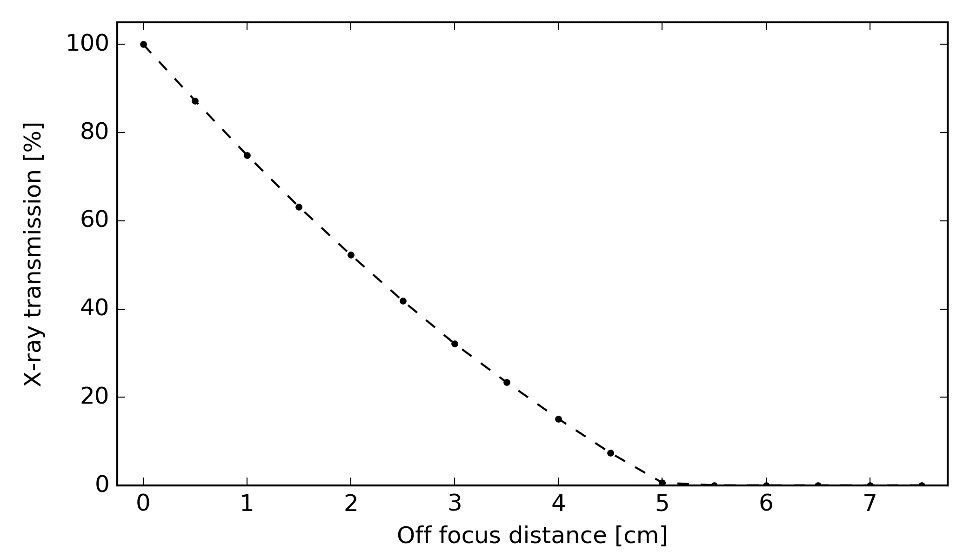


**Figure 1:** The relative transmission of x-ray photons as a function of the off-focus distance of the x-ray tube in relation to the focal point of the cone beam collimator.


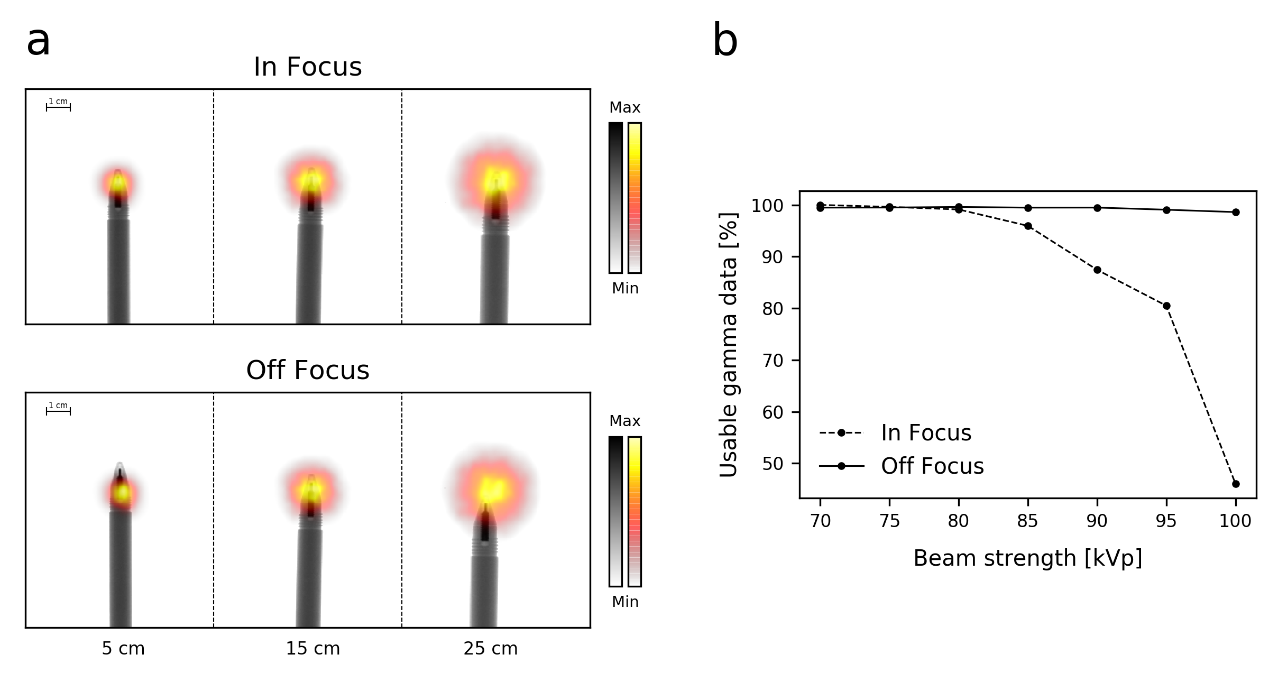


**Figure 2:** (a) The fluoroscopic (in gray-scale) and nuclear (in color) projections obtained with the in-focus and off-focus x-ray tube configurations. (b) The usable gamma data when simultaneously acquiring nuclear and fluoroscopic projections, as a function of beam strength, with the in-focus and off-focus x-ray tube configurations.
